# Supplementary material for: A DR/NIR Hybrid Polymeric Tool for Functional Bio-Coatings: Theoretical Study, Cytotoxicity, and Antimicrobial Activity
Source: Polymers (Basel). 2023 Feb 10;15(4):883. doi: 10.3390/polym15040883 (PMC9967255; doi:10.3390/polym15040883)
Supplement: Supplementary file 1 [file polymers-15-00883-s001.zip › polymers-2174252-supplementary.pdf]

A DR/NIR Hybrid Polymeric Tool for Functional Bio-Coatings: Theoretical Study, Cytotoxicity, and Antimicrobial Activity

Rosita Diana [1], Francesco Silvio Gentile [2], Simona Concilio [2,3], Antonello Petrella [2], Raffaella Belvedere [2], Martina Schibeci [4], Angela Arciello [4,5], Luigi Di Costanzo [1] and Barbara Panunzi [1,*]

[1] Department of Agricultural Sciences, University of Naples Federico II, 80055 Napoli, Italy
[2] Department of Pharmacy, University of Salerno, 84084 Salerno, Italy
[3] Bionam Research Center for Biomaterials, University of Salerno, 84084 Salerno, Italy
[4] Department of Chemical Sciences, University of Naples Federico II, 80126 Napoli, Italy
[5] Istituto Nazionale di Biostrutture e Biosistemi (INBB), 00136 Roma, Italy
* Correspondence: barbara.panunzi@unina.it

SUPPLEMENTARY MATERIAL

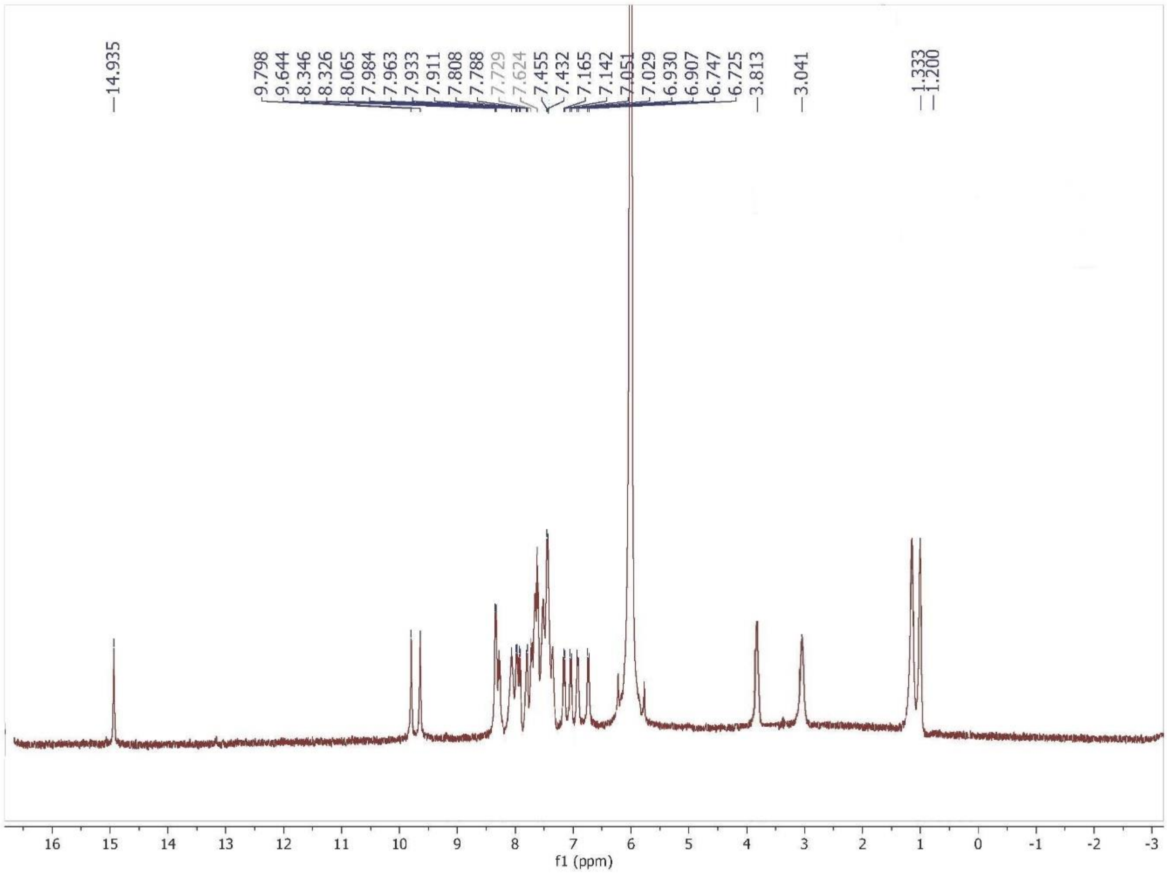

Figure S1. Hard copy ^{1}H NMR (500 MHz, TCE–d2, 25 °C, ppm) of ZnNB.

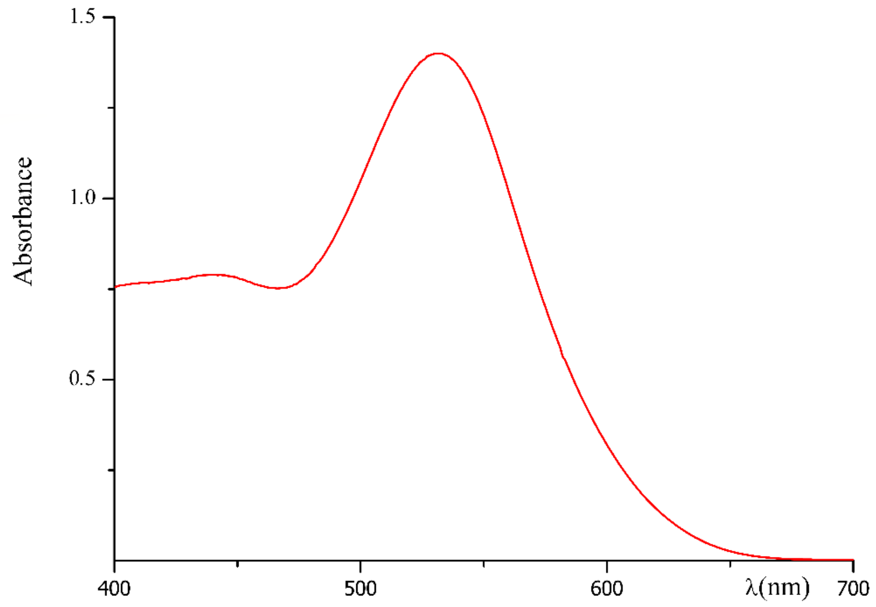

Figure S2. Absorbance spectrum of ZnNB deposed as powder crystals onto a quartz slide.

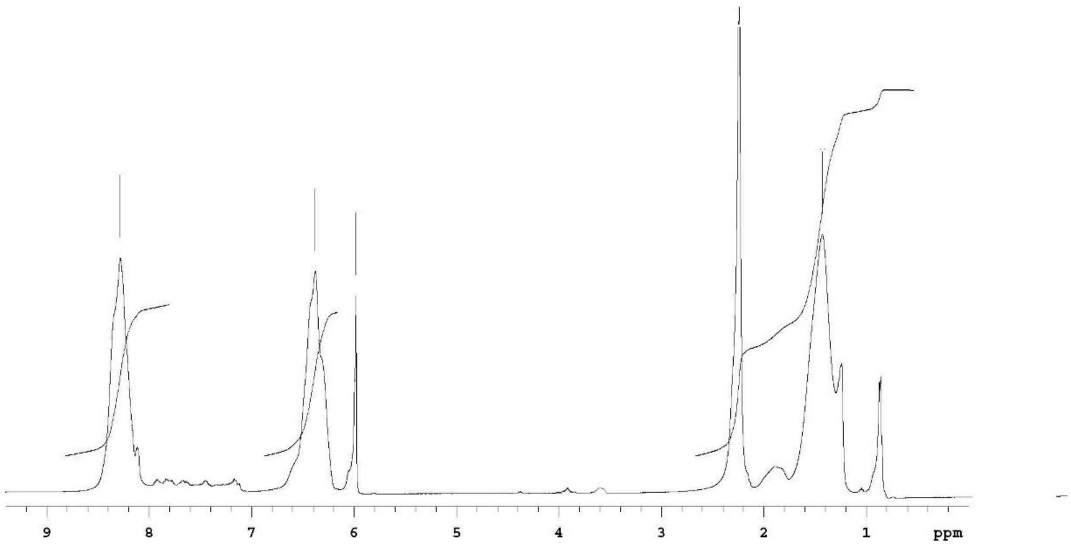

Figure S3. Hard copy ^{1}H NMR (500 MHz, TCE-d2, 25 °C, ppm) of P4VP-ZnNB 20%.

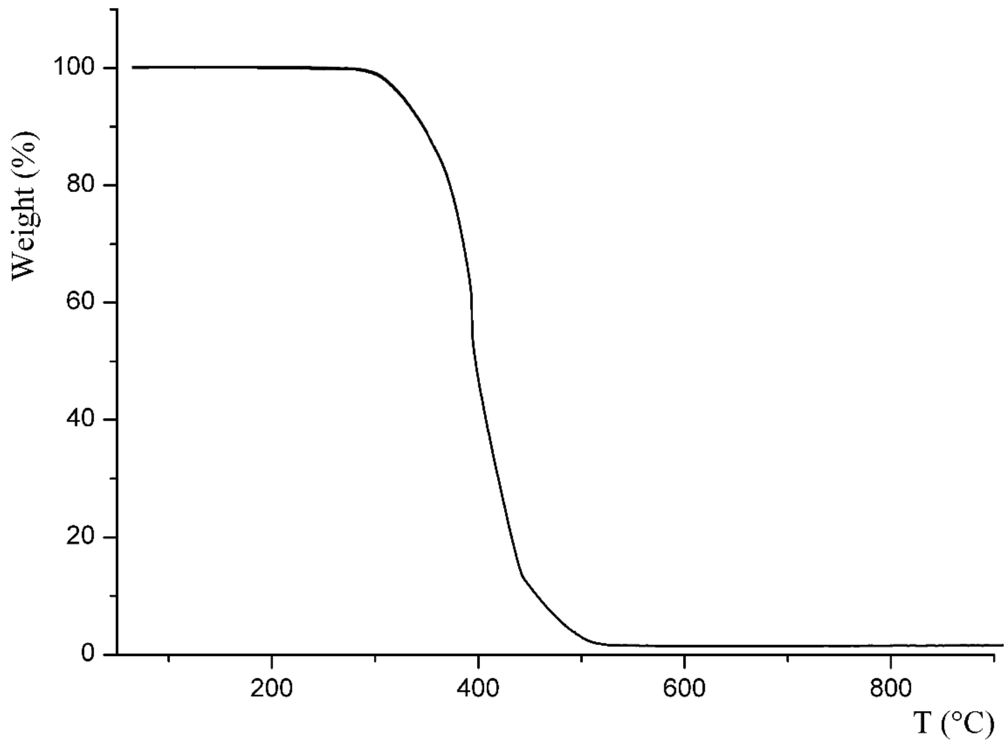

Figure S4. TGA curve of P4VP-ZnNB 20%.

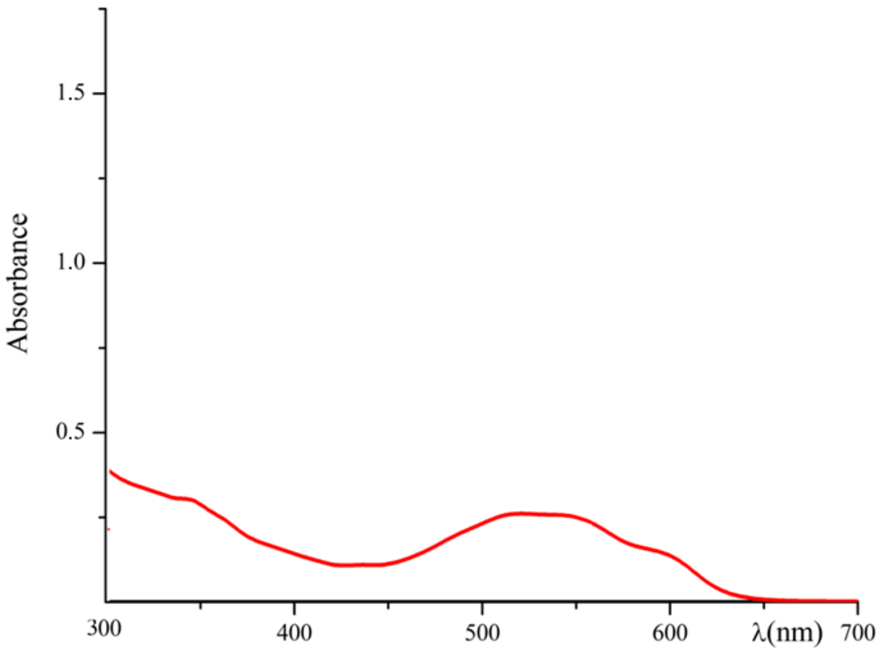

Figure S5. Absorbance spectrum of a thin transparent film P4VP-ZnNB 20% spin-coated onto a quartz slide.

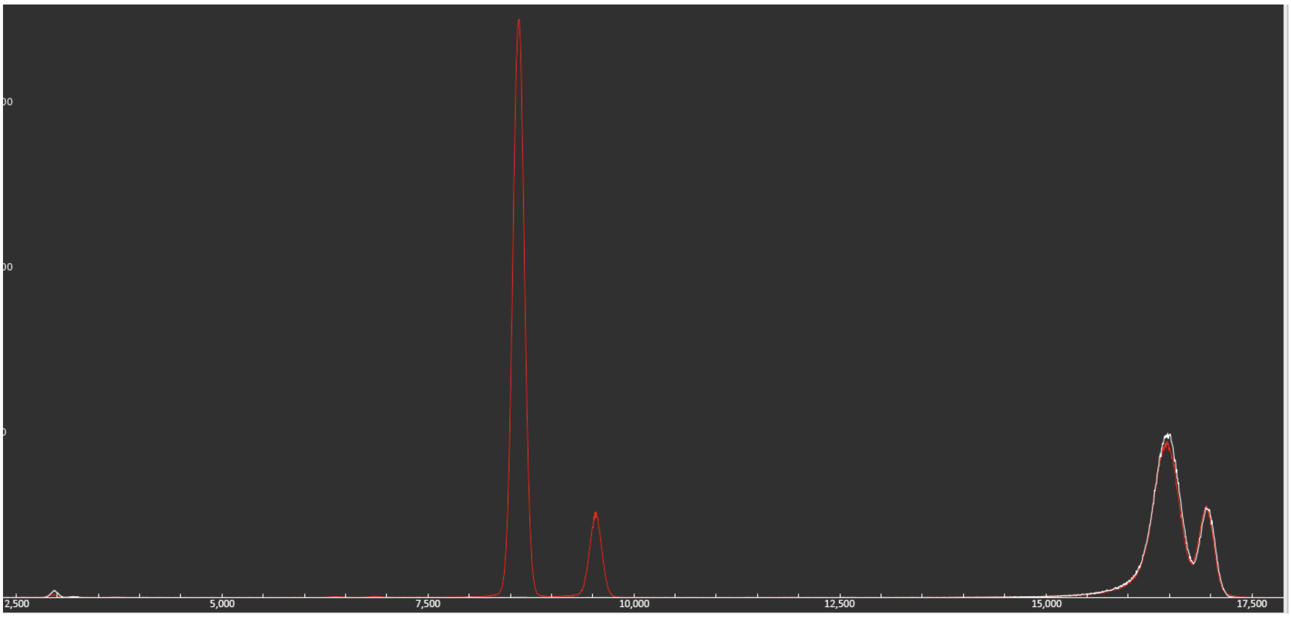

Figure S6. Energy spectrum obtained from X-ray fluorescence measurement of washed single crystal of ZnNB shows characteristic K edge (8.6 keV) and K peaks of zinc (red color). For comparison, the spectrum obtained for Kapton (shown in white color) is included. The material was used as support and a reference material.

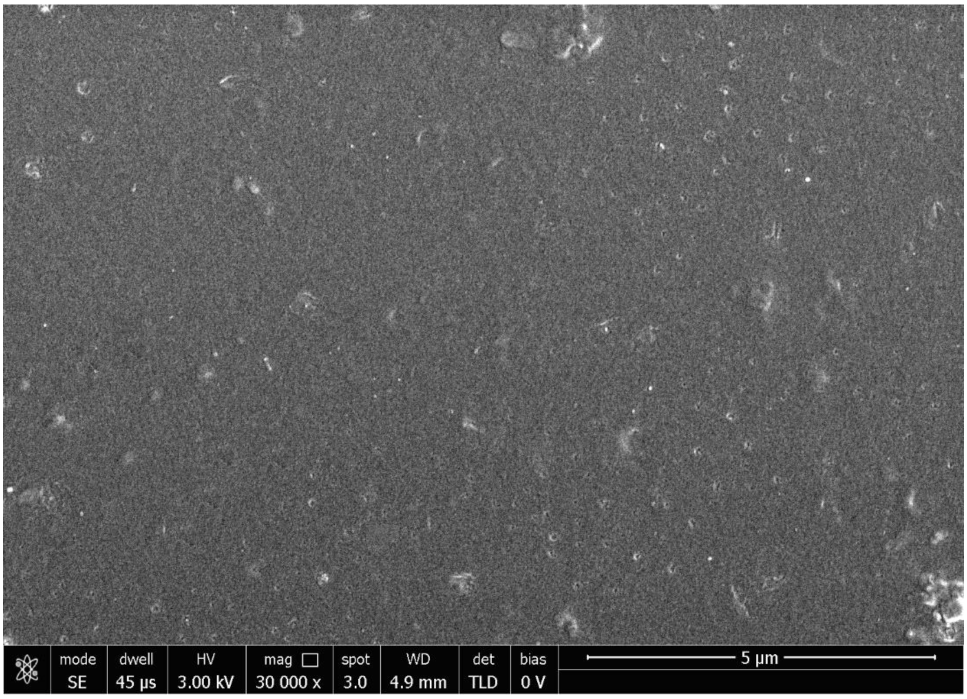

Figure S7. SEM micrographs of P4VP-ZnNB 20% film deposed onto quartz slide and metalized by gold sputtering (about 5 nm)

Table S1. Data collection statistics for crystals of ZnNB

Color	Red
Temperature (K)	100
Wavelength (Å)	0.619
Crystal system	Monoclinic
Space group	$P\ 2_1/n$
a (Å)	16.442 (7)
b (Å)	4.790 (2)
c (Å)	25.880 (9)
α (°)	90.00 (3)
β (°)	91.24 (11)
γ (°)	92.49 (3)
Resolution (Å)	2.32−0.78
R−merge	0.053
I/s(I) (highest shell)	14.14 (2.07)
Completeness (%)	99.4
Multiplicity	6.5 (5.5)
Mosaicity (°)	0.281
Volume	5628 $Å^3$
Z	1
